# Feasibility and Preliminary Outcomes of a Mobile Intervention Combining Cognitive Behavioral Therapy, Virtual Coaching, and Nicotine Replacement Therapy for Nicotine Vaping Cessation

**DOI:** 10.1089/tmr.2023.0009

**Published:** 2023-04-19

**Authors:** Jamie Webb, Yu-Ting Lin, Alfonso Ang, Darcy Michero, Azeem Majeed, Andreas Eisingerich, Suzette Glasner

**Affiliations:** ^1^ Department of Clinical Affairs, Digital Therapeutics, Inc., San Francisco, California, USA.; ^2^ School of Marketing, University of New South Wales, Sydney, Australia.; ^3^ Imperial College Business School, Imperial College London, London, United Kingdom.; ^4^ UCLA Integrated Substance Abuse Programs, Los Angeles, California, USA.; ^5^ School of Medicine, Imperial College London, London, United Kingdom.

**Keywords:** cognitive behavioral therapy, vaping, nicotine replacement therapy, mHealth

## Abstract

**Background:**

Despite research demonstrating that those who use e-cigarettes, also known as vaping, express an interest in quitting, evidence-based vaping cessation interventions are lacking. The purpose of this study was to examine the feasibility and preliminary outcomes of an mHealth vaping cessation intervention.

**Methods:**

Adults (*N* = 51) who were vaping nicotine were recruited online and enrolled in a 6-week mHealth intervention combining nicotine replacement therapy (NRT), self-guided cognitive behavioral therapy (CBT), and coaching support through telephone and asynchronous messaging. Feasibility and self-reported 7- and 30-day abstinence were assessed at baseline and 1-month postquit date.

**Results:**

The majority of participants completed treatment (45/51) and found the intervention helpful in supporting their vaping behavior change objectives. At 1-month postquit date, 48.9% (22/45) of study completers reported 7-day point prevalence abstinence and 28.8% (13/45) reported continuous 30-day abstinence.

**Conclusions:**

Findings provide preliminary support for an mHealth intervention approach to vaping cessation combining remote CBT-based coaching with NRT.

## Introduction

Despite the declining global prevalence of smoking tobacco over the past several decades, the use of new tobacco products such as e-cigarettes has increased, particularly among young adults.^1,2^ Vaping has been listed as a growing global public health concern, with vaping sales predicted to double by 2023 and a call for the development of safe and effective vaping cessation interventions in the 2020 U.S. Surgeon General's Report on Smoking Cessation.^3,4^ Nevertheless, only a handful of peer-reviewed research reports on vaping cessation have been published, despite the observation that ∼90% of long-term vapers report being dependent on e-cigarettes, and >60% of Americans who use e-cigarettes express the intention to quit for good.^5,6^

Delivery of evidence-based smoking cessation interventions using digital platforms has numerous advantages over traditional approaches, including ease of accessibility, delivery of real-time feedback to users, scalability, and cost-effectiveness.^7^ Given that more than a third of all smokers turn to the internet for smoking cessation support each year, coupled with widespread mobile phone access globally, digital approaches to vaping cessation could offer a promising solution.^8,9^ A few studies to date have leveraged technology to deliver evidence-based interventions targeting vaping behavior.

These include investigations of vaping cessation support and skill building through text messaging^10^ and brief contingency management (i.e., financial incentives contingent on biochemically verified vaping abstinence) delivered through a smartphone app for 28 days.^11^ Despite the promise of these approaches, the integration of pharmacotherapy and evidence-based behavioral treatment through a telemedicine modality has not yet been examined as an intervention strategy for vaping cessation. In efforts to bridge this gap, this study examined the feasibility, preliminary outcomes, and user experiences of a novel mHealth intervention combining nicotine replacement therapy (NRT) with digital cognitive behavioral therapy (CBT) and human-delivered coaching support.

## Methods

### Participants

Participants were 51 adults who currently vape nicotine. The study was approved by the Imperial College London Research Ethics Committee (ICREC reference 20IC6306). Participants were recruited through social media (i.e., Facebook) and screened using an online survey.

Individuals who met the following eligibility criteria were then given a complete description of the study and informed consent was obtained: (1) age ≥21 years, (2) a U.S. resident, (3) self-reported current e-cigarette use (vaping nicotine in the past 30 days), (4) interest in changing vaping behavior, (4) sufficient phone functionality to utilize the app, and (5) English speaking. Participants were excluded if they (1) smoked cigarettes in the past 30 days, (2) were currently using any other form of nicotine cessation, (3) had used the mHealth app under study previously, and (4) had a serious or unstable medical condition that made study participation difficult.

### Procedure

After online screening, eligible participants who provided informed consent were provided with a link via e-mail to complete baseline questionnaires online. Participants were then given instructions to download the mHealth app and advised to set a target date to quit or reduce vaping within 2 weeks. At 1-month post-target-quit-date, participants were invited to complete the online follow-up assessment through e-mail. The total length of trial participation was ∼6 weeks. Participants were compensated $20 after completion of each baseline and follow-up assessments.

### Intervention

Quit Genius-Vaping (QG-V), a commercially available mHealth tobacco cessation program (Digital Therapeutics, Inc., San Francisco, CA), combines NRT with a smartphone application containing self-guided CBT content, and concurrent support from a quit coach who provides asynchronous messaging to reinforce CBT skills practice. To tailor the pace, feedback, and digital CBT content to the individual, the application collects user data including app utilization, program completion, motivations for quitting vaping, and quit date. Based on these variables, personalized content is delivered in the form of personalized quizzes, CBT skills training exercises, and personalized progress feedback.

A certified tobacco cessation coach provides asynchronous messaging to reinforce CBT skills through a digital chat interface. Drawing from the extended CBT model developed by Hall et al., content areas within the app include (1) motivation, (2) dependence and withdrawal, (3) social support, (4) identifying triggers, (5) coping with cravings, (6) managing negative affect, (7) weight gain, and (8) relapse prevention.^12^ Digital CBT content is divided into two stages. Stage 1 (“Essentials”) comprises psychoeducation and motivational exercises, including preparing for the quit date, information about how and why to use NRT, and articulating reasons for quitting.

Stage 2 (“Sustain”) is introduced postquit date, and focuses on relapse prevention, self-monitoring by logging triggers, cravings, and daily vaping behavior. After quitting vaping, participants are prompted to log their vaping status upon opening the app. Subsequently, tailored feedback is delivered concerning abstinence status and associated health and financial benefits throughout the active intervention phase.

All participants received a 30-min telephonic intervention with their quit coach, who was certified by the National Centre for Smoking Cessation and Training. During this call, participants were introduced to QG-V, and discussed their individualized quit plan with their coach, including preparation for their target date, orientation to the various forms of NRT provided as part of the study, and how to use them. All interactions with the coach thereafter were conducted through the in-app chat platform. Participants enrolled in the trial were assigned to one of two study quit coaches, with equivalent caseloads assigned to each.

Once assigned, participants interacted with the same coach throughout the active intervention phase of the trial. Using the app, participants could (1) monitor their progress toward their vaping goals, including health improvements and financial benefits and (2) access to a “Craving Toolbox” with audio-delivered therapeutic exercises (e.g., mindfulness, meditation, and breathing) for coping with vaping urges.

#### Nicotine replacement therapy

All participants were given over-the-counter NRT (2 or 4 mg gum and/or 16 or 24 h patches) for 6 weeks, with the first 2-week supply mailed at baseline. Participants were also allowed to use other forms of oral NRT.

### Measures

Measures were gathered using online questionnaires at baseline and 1 month postquit date, and included self-reported days and minutes per day spent vaping in past 30 days; e-cigarette dependence scale (EDS) score, a 4-item scale (mean score range: 0–4) used to assess nicotine dependence among individuals who use e-cigarettes;^13^ and user satisfaction. Engagement with QG-V app comprised app log-ins, minutes on app, and messages to and from the quit coach.

### Statistical analysis

Statistical analyses were conducted with SAS version 9.4 software (SAS Institute, Cary, NC). Changes in outcome variables from baseline to 1 month post-QD were assessed using data from the 45 participants who completed the study, using paired-sample *t* tests for continuous measures and chi-square tests for binary measures. Statistical significance was set at *p* < 0.05.

## Results

A total of 99 individuals were screened, of whom 37 were ineligible. Of the 62 eligible individuals, 11 failed to complete their baseline visit. The remaining 51 eligible participants enrolled into the study. Participants were, on average, 27.9 (SD = 8.1) years of age (range: 21–46), predominantly female (54.6%), Caucasian (68.2%), and college educated (68.2%). On average, participants had been vaping for 3.1 years (SD = 1.7), with an average EDS score of 10.1 (SD = 3.0). Most participants (70.5%, *n* = 36) had made previous quit attempts.

By the quit date (an average 8 days after app download), 82.3% (42/51) had logged into the app. At the 1-month postquit date, 50.9% (26/51) were continuing to log into QG-V. Over the course of the study, on average, participants opened the app 25.2 times (SD = 31.8), spent 74.2 min in the app (SD = 85.2), sent 7.3 (SD = 8.6) messages to their coach, and received 12.4 (SD = 8.2) messages from their coach. Treatment completion, defined as having attended the 1-month postquit date data collection visits, was achieved by the majority of study participants (88%).

Perceived helpfulness of the app intervention was assessed using a consumer feedback questionnaire used in prior intervention trials evaluating mobile apps targeting tobacco cessation.^14^ At 1-month postquit date, on a scale from 0 to 3, with corresponding ratings of “poor, neutral, good, or excellent,” 87% (39/45) of participants rated the quality of service they received through QG-V as good or excellent, 84% (37/45) reported that most or all of their needs were met by QG-V, and 98% (43/45) rated the program as helpful or very helpful. Over 91% of participants reported that they would resume the intervention if they needed help with vaping in the future, 87% reported that the intervention helped them to effectively quit vaping, and 93% would recommend QG-V to a friend.

The most helpful components of the intervention comprised the quit coach (13/45, 28.8%) and the digital CBT content (12/45, 26.7%), whereas only 6.6% of participants rated the self-monitoring section as the most helpful aspect.

### Vaping

At 1-month post-QD, 48.9% (22/45) of study completers (88% of the sample) reported that they were abstinent from vaping for the past 7 days and 28.8% (13/45) reported that they were abstinent from vaping for the past 30 days. Of those who reported that they had vaped in the past 30 days, there was a significant reduction in the number of days of vaping in the past 30 days from baseline (*M* = 24.7, SD = 8.7) to 1 month post-QD (*M* = 3.9, SD = 2.0), *t*(30) = 13.2, *p* < 0.001. Likewise, duration (i.e., minutes per day) of vaping in the past 30 days declined from baseline (*M* = 75.8, SD = 37.5) to 1 month post-QD (*M* = 26.5, SD = 31.8), *t*(30) = 4.4, *p* < 0.001. On the EDS, a corresponding reduction in nicotine dependence severity was observed from baseline (*M* = 2.5, SD = 0.8) to 1 month post-QD (*M* = 1.5, SD = 0.9), *t*(44) = 6.5, *p* < 0.001.

## Discussion

This pilot study showed that providing vaping cessation support using a digital CBT intervention, combined with human-delivered coaching and NRT, is a feasible, acceptable, and potentially efficacious approach to addressing e-cigarette use. The QG-V app was rated as highly acceptable and well used by the majority of participants until their quit date, with half continuing to use it 1-month post-QD. In addition, participants were highly engaged with their quit coaches through asynchronous messaging throughout the study, highlighting the value of both the digital and human coach-facilitated integrated components of the intervention.

Preliminary vaping outcomes were promising, with reductions in e-cigarette dependence severity and corresponding changes in e-cigarette use from baseline to 1-month post-QD. Continuous self-reported abstinence from vaping over 30 days was observed in nearly a third of participants, with those who continued vaping demonstrating substantial reductions in vaping frequency.

Several limitations warrant comment. First, biochemical verification of abstinence was not conducted given the challenges of doing so in digital cessation trials.^15^ Second, in the absence of a control condition, efficacy of the digital and coach-delivered behavioral therapy components of QG-V over and above NRT cannot be ascertained. Third, although the study timeframe provides preliminary evidence concerning the short-term outcomes of this approach, whether the observed changes in vaping behavior extend into a longer term than 30 days is as yet unknown. Finally, given the elevated rates of vaping among youth of ages 13 to 17 years, extension of this approach to teens in future studies is important.

Studies of technology-assisted approaches to nicotine vaping cessation to date have focused largely on automated behavioral support, with limited use of evidence-based intervention strategies.^16^ This study advances the current literature on nicotine vaping cessation approaches, demonstrating the feasibility of delivering combination treatments integrating pharmacotherapy with evidence-based behavioral therapy using a smartphone application.

In conclusion, preliminary positive results from this single-arm pilot study suggest that QG-V warrants testing in a fully powered randomized controlled trial. Moreover, to facilitate a better understanding of the characteristics of populations for whom a digital vaping intervention approach is best suited, predictors of treatment response should be an additional focus of future studies. Given the paucity of validated vaping cessation interventions, these findings add direction to inform the development of scalable approaches to mitigate e-cigarette use.

## Abbreviations Used

CBT: cognitive behavioral therapy
EDS: e-cigarette dependence scale
NRT: nicotine replacement therapy
QG-V: Quit Genius-Vaping
